# “Smart” RCTs: Development of a Smartphone App for Fully Automated Nutrition-Labeling Intervention Trials

**DOI:** 10.2196/mhealth.5219

**Published:** 2016-03-17

**Authors:** Ekaterina Volkova, Nicole Li, Elizabeth Dunford, Helen Eyles, Michelle Crino, Jo Michie, Cliona Ni Mhurchu

**Affiliations:** ^1^ National Institute for Health Innovation School of Population Health University of Auckland Auckland New Zealand; ^2^ Food Policy Division The George Institute for Global Health University of Sydney Sydney Australia; ^3^ Carolina Population Center The University of North Carolina at Chapel Hill Chapel Hill, NC United States; ^4^ Epidemiology and Biostatistics School of Population Health University of Auckland Auckland New Zealand

**Keywords:** randomized controlled trial, smartphone, public health, nutrition labeling

## Abstract

**Background:**

There is substantial interest in the effects of nutrition labels on consumer food-purchasing behavior. However, conducting randomized controlled trials on the impact of nutrition labels in the real world presents a significant challenge.

**Objective:**

The Food Label Trial (FLT) smartphone app was developed to enable conducting fully automated trials, delivering intervention remotely, and collecting individual-level data on food purchases for two nutrition-labeling randomized controlled trials (RCTs) in New Zealand and Australia.

**Methods:**

Two versions of the smartphone app were developed: one for a 5-arm trial (Australian) and the other for a 3-arm trial (New Zealand). The RCT protocols guided requirements for app functionality, that is, obtaining informed consent, two-stage eligibility check, questionnaire administration, randomization, intervention delivery, and outcome assessment. Intervention delivery (nutrition labels) and outcome data collection (individual shopping data) used the smartphone camera technology, where a barcode scanner was used to identify a packaged food and link it with its corresponding match in a food composition database. Scanned products were either recorded in an electronic list (data collection mode) or allocated a nutrition label on screen if matched successfully with an existing product in the database (intervention delivery mode). All recorded data were transmitted to the RCT database hosted on a server.

**Results:**

In total approximately 4000 users have downloaded the FLT app to date; 606 (Australia) and 1470 (New Zealand) users met the eligibility criteria and were randomized. Individual shopping data collected by participants currently comprise more than 96,000 (Australia) and 229,000 (New Zealand) packaged food and beverage products.

**Conclusions:**

The FLT app is one of the first smartphone apps to enable conducting fully automated RCTs. Preliminary app usage statistics demonstrate large potential of such technology, both for intervention delivery and data collection.

**Trial Registration:**

Australian New Zealand Clinical Trials Registry ACTRN12614000964617. New Zealand trial: Australian New Zealand Clinical Trials Registry ACTRN12614000644662.

##  Introduction

Smartphone technology offers promising new ways to deliver health interventions and undertake research [1], and smartphone-assisted randomized controlled trials (RCTs) are an emerging methodology. The role of smartphone technology in RCTs varies from provision of simple information or text message reminders to participants to more complex tools enabling, for example, self-monitoring or data collection [2-4]. Messages have been the most common tool used in smartphone-assisted studies to date, although specialized smartphone apps are increasing in popularity [5]. Although systematic reviews suggest mixed evidence of the effectiveness of smartphone-delivered health interventions compared to traditional methods [6,7], advantages of such programs include ability to deliver an intervention remotely and a potentially wider population reach. Greater participant retention and adherence to the intervention and improved convenience for participants compared to traditional methods have also been reported for smartphone-assisted interventions [2,8,9].

Most smartphone RCTs to date have been only partially technology-assisted, that is, smartphone technology is used as an add-on to an existing behavior change program or an automated intervention is compared with a standard technology-free control group [3,10]. Automated RCTs conducted entirely via a smartphone app are a novel approach in health research, and thus, limited published data are currently available in this field. To date, we are aware of only 1 other RCT (a smoking cessation app) that used this approach [11].

Here we describe a new smartphone app developed for use in automated RCTs on the effects of different nutrition label formats on the healthiness of consumer food purchases in 2 countries (Australia and New Zealand) [12,13]. The app (Food Label Trial, FLT) was designed to overcome 2 common challenges of nutrition-labeling interventions: (1) delivery of various nutrition label formats for foods in real-world supermarkets (the intervention) and (2) collection of reliable, objective, household-level food shopping data.

Although the potential public health benefits of interpretive, easy-to-understand nutrition labels are generally accepted [14], it is difficult to test the effectiveness of such labels as an intervention in real-world retail settings. Therefore, assessment of their effectiveness is often only possible in controlled settings [15] or in nonrandomized natural experiments [16]. Technology used in the FoodSwitch smartphone app [17], available in Australia and New Zealand, enables delivery of nutritional information via digital nutrition labels. FoodSwitch users place their smartphone camera over the barcode of a packaged food, the unique barcode is then matched with a product in the underlying food database, and an interpretive nutrition label for the scanned product is displayed on screen. The intervention delivery mode of the FLT app uses the same technology to deliver a nutrition-labeling intervention to trial participants.

A second challenge is collection of individual-level data on food purchases. Traditional methods involve collection of itemized food shopping receipts. However, this requires manual coding and data entry, which is time-consuming and resource intensive [18-20]. Other ways of recording individual purchases, for example, via barcode scanners [21], have also been explored but have presented limitations such as the need for additional equipment or being limited to specific participating stores. To overcome this challenge, our RCT app was designed to have inbuilt data collection functionality using barcode scanning technology.

The aim of this paper is to describe the development and functionality of the smartphone app used for the trials, provide an overview of the end product, and report preliminary usage statistics and common technical issues.

##  Methods

### Approvals

Both trials received appropriate ethics approvals (University of Sydney Human Research Ethics Committee, reference number 460; University of Auckland Human Participant Ethics Committee, reference number 011390).

### Trial Overview

**Figure 1:**
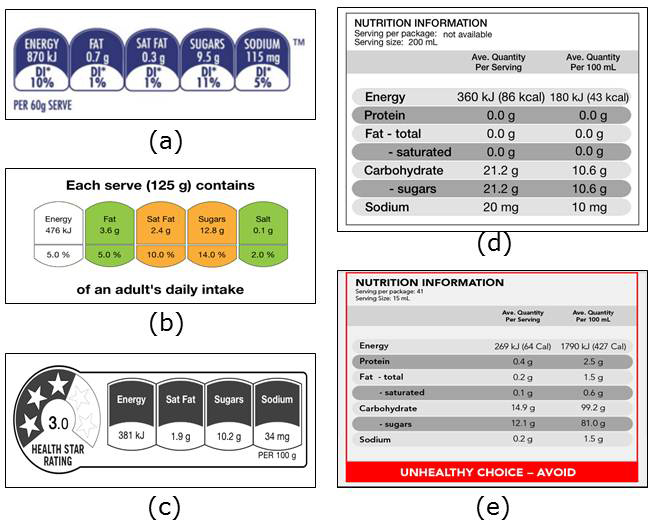
Nutrition-labeling formats allocated to intervention and control groups in the trials. (a) Daily Intake Guide*. (b) Traffic Lights. (c) Health Star Rating. (d) Nutrition Information Panel (control arm). (e) Warning Label*. *Australian trial only.

Full trial protocols, including aims, design, outcome measurements, and power calculations, have been previously published [12,13]. In summary, the randomized controlled trials aimed to assess the effects of different nutrition label formats on the healthiness of consumer food purchases. Individual food and beverage shopping data were collected during 5 weeks (1-week baseline and run-in and a 4-week intervention period). Eligible participants were randomized to 1 of 5 nutrition-labeling formats in Australia and 1 of 3 labeling formats in New Zealand (Figure 1): Daily Intake Guide [22] (Australian trial only), Traffic Light labels [23], Health Star Rating [24], Nutrition Information Panel (control) [25], or a Warning Label [13] (Australian trial only). Both intervention and control nutrition labels were delivered via the smartphone app to minimize technology bias.

### Development of the App

#### Key Requirements

The app had 3 key functionality requirements: (1) fully automating the conduct of RCTs [12,13], that is, consent, screening, randomization, and questionnaire administration; (2) delivering trial intervention, that is, nutrition labels; and (3) facilitating data collection.

**Figure 2:**
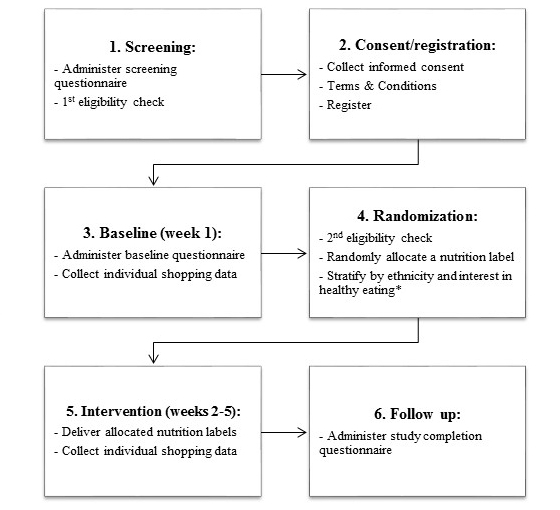
Sequence of the key trial events automatically delivered via the FLT mobile app. *New Zealand trial only.

The framework for the first function is presented in Figure 2 as a sequence of the key trial events. Progression through those events was determined by in-app and server-side checks, including an email address check to prevent duplicate registrations, a check that consent has been provided and terms and conditions have been accepted, eligibility at screening, and a check that at least 15 barcoded food or beverage items were recorded during week 1 (run-in phase requirement). A timeline check (from the moment of registration) was also required to ensure delivery of key events such as randomization (end of week 1) and the follow-up questionnaire (end of week 5).

Intervention delivery functionality provided users with a nutrition label for a scanned food product. The format of the label provided was determined by the participant’s randomization allocation (Figure 1). Designs of digital nutrition labels in the app were based on the relevant style guides [22-25]. Similar to the FoodSwitch app, nutrient content information for the scanned product was obtained by matching the barcode number with one from the food composition database at the back end.

Data collection functionality used barcode scanning to create electronic itemized records of food purchases made by participants, and the smartphone camera was used to photograph the till receipts. Participants were requested to record all food purchases during the 5-week trial period. Additional information on demographics and usual shopping patterns was collected via in-app baseline and follow-up questionnaires. Two in-app tutorials were developed for user training purposes. In-app and push notifications and reminders were developed to prompt user engagement and adherence to the trial protocol and timelines. The following ethical and security requirements were adhered to: (1) A participant information statement was available to participants via the app throughout the trial, and (2) all data collected via the app were stored securely with identifiable information stored separately from trial outcome data.

Two similar versions of the FLT app were created for the Australian and New Zealand RCTs, with the following differences between the apps for the two countries: (1) five intervention arms in the Australian app versus 3 for New Zealand; (2) different randomization algorithms with (New Zealand) or without (Australia) stratification by ethnicity and self-reported interest in healthy eating [12]; (3) Australian and New Zealand trial-specific participant information and consent statements, terms and conditions, logos, and content-management systems; and (4) country-specific back-end food composition databases.

#### Backend Food Composition Databases

Country-specific food composition databases provided the nutrient information to create nutrition labels and assess outcome measures. The Australian version of the app used The George Institute for Global Health’s FoodSwitch database [17], which currently contains nutrition information for more than 65,000 Australian packaged food items. The New Zealand version of the app used the NZ FoodSwitch database, which currently contains nutrition information for more than 21,000 products. The most up-to-date versions of the food composition datasets available at the time of app development were used.

#### Usability Evaluation

The initial decision on whether an FLT smartphone intervention was feasible to conduct in Australia and New Zealand was based on the popularity of the FoodSwitch app, which currently has more than 600,000 downloads in Australia (population 23.7 million in 2015) and 65,000 downloads in New Zealand (population 4.5 million in 2015). Because the FoodSwitch and FLT apps’ core functionality were very similar, separate usability evaluation was not deemed necessary for the FLT app.

#### Development and Testing

The app was designed by research teams at used The George Institute for Global Health, University of Sydney, Australia, and the National Institute for Health Innovation, University of Auckland, New Zealand. Software and interface development was led by Buzinga Apps, Australia. The app was developed for Android and iOS platforms, the most common smartphone platforms in Australia and New Zealand [17]. During the development period, weekly online conference meetings were held between the used The George Institute for Global Health, National Institute for Health Innovation, and Buzinga teams. The time frame for development was approximately 9 months, with the framework, interface, technical requirements, and algorithms being developed over the first 5 months (Jan-May 2014) and the iterative development and testing process implemented over the following 4 months (June-Sep 2014). All components of the FLT app and the final versions were pretested by the software developer and research teams. Final products, for both Android and iOS, were tested by independent volunteers from the same research institutions who were not involved in the project. Final versions of the FLT app were submitted to Apple and Android app stores in October 2014. The initial version of the app was compatible with smartphones running either iOS 7 and above or Android 4.3-4.4. The app was updated as new versions of the operating systems became available.

#### Data Management

All data collected via the FLT app were automatically transmitted to a trial database located on a remote server. Country-specific content management systems were developed for data processing, management, and extraction. Automatic in-app and server-side logic checks on the incoming data were used to ensure adherence to trial requirements and progress through the stages.

#### Technical Issue Management

On-going quality control of the collected data was carried out by research teams to identify any potential issues. Any identified issues were prioritized by the research team and addressed by Buzinga Apps based on the impact on trial data and the level of inconvenience to participants. Weekly meetings were held between researchers and the app development teams to ensure timely resolution of major technical problems. Two updates of the app for the Android platform and 1 for iOS platform were released after the initial app launch to provide fixes for identified issues.

### Analysis of the App Usage

Data collected from the trial app between October 2014 and June 2015 were used for analyses and represent the first nine months of the trial. Simple descriptive statistics were used to report the number of downloads, registrations, randomizations, and the amount of individual shopping data collected via the app. The final trial completion rates will be reported in the results papers (the trials were ongoing at the time of drafting).

## Results

### Functionality Overview

**Figure 3:**
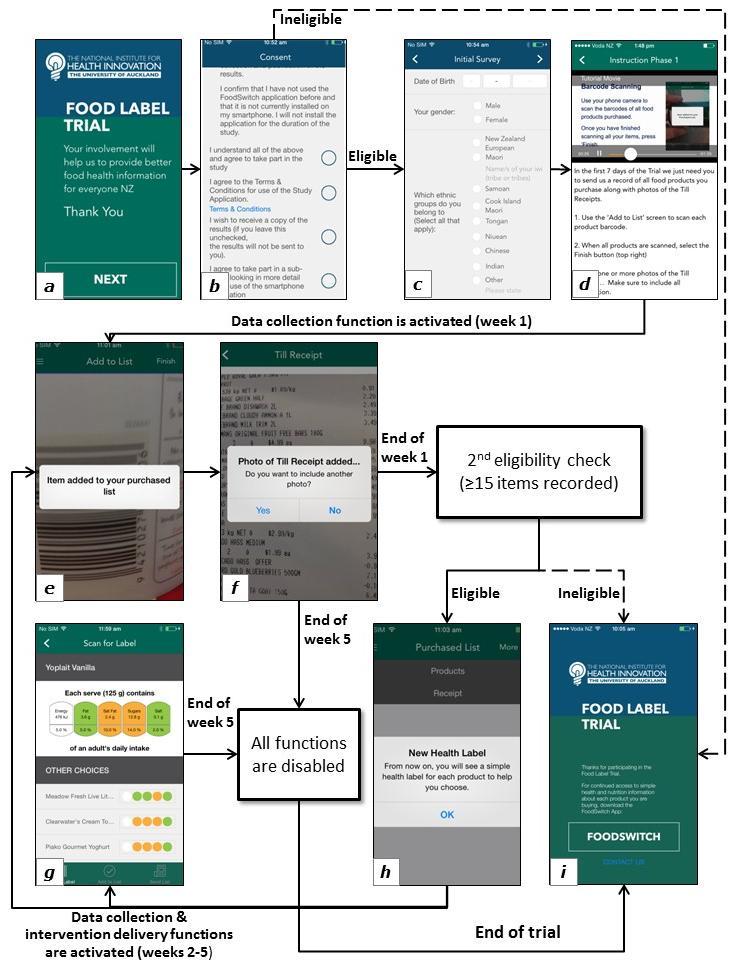
Food Label Trial app key functionality flowchart with sample screenshots (from the New Zealand version of the app). (a) Welcome screen. (b) Consent and terms and conditions screen. (c) Baseline questionnaire screen. (d) Tutorial. (e) Data collection mode: barcode scanning. (f) Data collection mode: adding a matching till receipt image. (g) Intervention delivery: traffic light label arm. (h) Postrandomization in-app notification. (i) End of trial screen, offering a link to the FoodSwitch app.

A flowchart with sample screenshots is presented in Figure 3, and the 16 main functionality components of the FLT app are summarized in Table 1. The first event in the FLT app was the informed consent and registration process. All subsequent events were automatically triggered either by a task completion (eg, a questionnaire) or by reaching a key trial time point (eg, end of baseline phase after 1 week). The trial tutorials are available in the Multimedia Appendices 1 and 2. The logic and schedule of key reminder messages and notifications are summarized in Table 2. The messages were triggered either by the trial timelines or user’s progress on task completion.

**Table 1:** Main functions of the Food Label Trial app.

Component	Time point	Description
Consent and T&C^a^ statement	User opens the app for the first time	On-screen consent statement with links to full PIS and T&C documents. Multichoice tick box answer options. Acceptance of the consent statement and T&C were mandatory.
Registration form	After screening questionnaire	Questions with free text answer options. A requirement to enter a unique email address.
Questionnaires	Screening: after consent Baseline: prior to week 1 Follow-up: end of week 5	Questions with multi-choice (tick-box or sliding scale) or free text answer options.
Eligibility checks	First: at screening Second: at the end of week 1	Eligibility was determined from the screening questionnaire answers (first check) and the number of products recorded during baseline (second check).
Randomization	End of week 1	Server-side central blocked randomization with variable block sizes.
Barcode scanning	Weeks 1-5	Barcode numbers of the scanned products were matched with an in-app backend food database.
Electronic lists of purchases	Weeks 1-5	Automatically generated electronic list of scanned product items
Till receipt capture	Weeks 1-5	Smartphone camera is engaged to photograph the till receipt images.
Intervention (nutrition label) delivery	Weeks 2-5	For recognized products, a nutrition label was displayed. The label format was determined by the user’s randomization allocation. A random selection of other products from the same category was displayed under the label.
Crowdsourcing: missing product information	Weeks 2-5	An optional functionality allowed users to submit photos of missing products (as with the crowdsourcing function of the FoodSwitch app [17].
Tutorials	First: before week 1 Second: before week 2	Short in-app video clips introducing the app functionality and the required tasks.
Reminders and notifications	Throughout the trial	Automatically triggered in-app and push notification messages. The logic and schedule are described in Table 2.
Progress tracker	Weeks 1-5	Showed the number of weeks completed on the trial.
History	Weeks 1-5	A history of all previously sent electronic lists
Trial information	Weeks 1-5	Information about the trial, research team and technical support contacts, PIS.
Link to FoodSwitch	Ineligible users, end of trial	A link to the FoodSwitch app offered as an alternative resource for healthy food choices.

^a^PIS: participant information statement, T&C: terms and conditions.

**Table 2:** Key notification and reminder messages delivered by the Food Label Trial app

Message description	Trigger	Time point and frequency	Type
Notification informing of ineligibility for the trial; offered a link to the FoodSwitch app.	First eligibility check failed	Once after the screening questionnaire	In-app message
Reminder to complete the registration or baseline questionnaire	Registration form not completed	Two days after consent, then once a week up to 4 times, then once a month until 5 weeks before the overall trial recruitment completion	Push notification
Reminder to record at least 15 items during week 1	User submitted a list of purchased products during week 1, and the total number of items recorded by the user to date is less than 15	After every submitted list that meets the criteria	In-app message
Notification that the product list has been successfully sent	User submitted a list of purchased products, and it was successfully transmitted to the trial database. The number of products recorded during week 1 is 15 items or more	After every submitted list that meets the criteria	In-app message
Reminder to record food purchases (baseline phase)	User has not sent any product lists since the beginning of week 1	At days 3 and 5 of week 1	Push notification
Reminder to keep recording food purchases (intervention phase)	User has not been sending through any new product lists	At days 4, 6, 9, 12, 18, and 24 since the last product list was sent OR since the beginning of the intervention phase	Push notification
Reminder that the trial is ending soon	Day 26 of the intervention phase	Day 26 of the intervention phase	Push notification
Request to complete the follow-up questionnaire	Day 28 of the intervention phase	Day 28 of the intervention phase	In-app message
Reminder to complete the follow-up questionnaire	User has not completed the follow-up questionnaire	Days 6, 12, 18, 24, and 30 from completion of the intervention phase	Push notification
Notification of the trial completion	User completed the follow-up questionnaire	Once after the follow-up questionnaire	In-app message

### Preliminary App Usage Statistics

**Figure 4:**
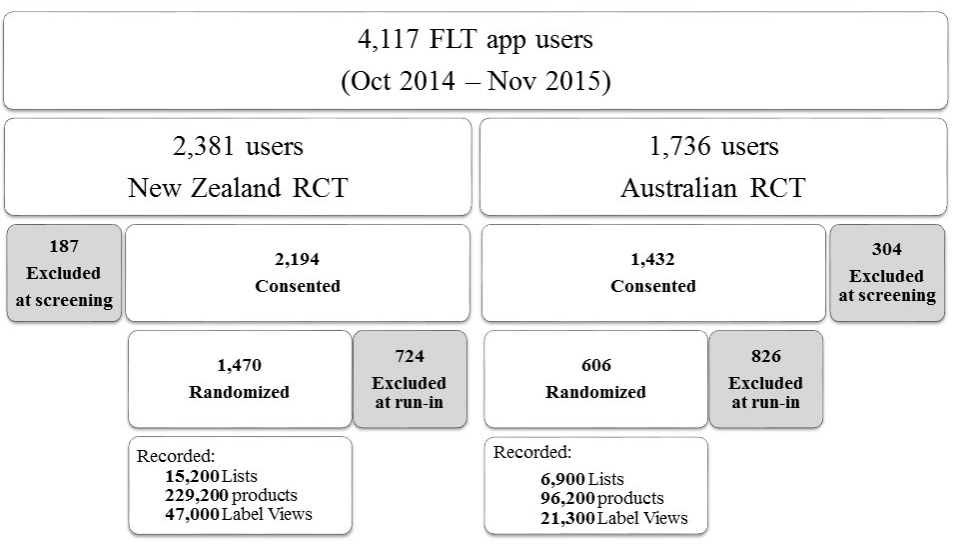
Food Label Trial app usage statistics for the period of October 2014-November 2015.

The FLT app usage statistics for the initial 13 months since the app launch are summarized in Figure 4 (October 2014-Nov 2015). In total, 3000 users downloaded the FLT app to undergo an eligibility check, and of these, close to 1500 unique users were randomized to receive the trial intervention. During this time, participants submitted close to 13,000 electronic shopping lists, with more than 150,000 individual food or beverage items and over 20,000 matching receipt images. The labeling intervention usage rates are reported in Figure 4 as a total number of “label views.”

### Key Technical Issues

The most common issues encountered were intermittent problems in connecting to the server while recording purchased products and reminder messages being sent very frequently. Other less-frequent issues, potentially caused by attempts to use the app on incompatible devices, included incorrect recording of consent status, problems viewing nutrition labels, registration issues, issues with focusing smartphone camera on barcodes during scanning, and multiple identical copies of baseline and follow-up surveys recorded for some users. A small number of users experienced issues accessing the trial intervention after the iOS 9.1 release in October 2015.

##  Discussion

The FLT app is a novel smartphone app designed to conduct an automated RCT on the effects of different nutrition labels on food purchases. To our knowledge, this is the first fully automated smartphone-based trial in public health nutrition. The use of the FLT app overcomes a major challenge commonly encountered in current nutrition-labeling interventions, that is, delivery of randomly allocated nutrition labels to study participants in their regular real-world food shopping locations. Previously, real-world effect of nutrition labels was assessed at a large scale in a natural experiment observation study [16]. Another large study used shelf-labeling in a selected chain of supermarkets [15]. However, a randomized controlled approach has not been possible, and as a result, the current trials are the first RCTs to measure the real-world effect of nutrition labels on food purchasing [26].

The FLT app enabled simplified collection of participant household food purchase data, automatically linked to demographic characteristics (via the participant ID number) and to the food database (via product barcodes). Compared to previous research in this area using shopping receipts as a primary data collection mode for food purchases [18-20], the FLT app substantially reduces the requirements for manual coding and data entry of packaged foods. To minimize potential bias due to technology influence, a control group nutrition label was also delivered via the smartphone, which replicates current mandatory nutrition labels found on the back of the pack of most food and beverage items in Australia and New Zealand.

Preliminary statistics of the FLT app usage have been promising; a large amount of data on intervention usage (label viewing) and individual food purchases has been collected to date. Completeness of electronic shopping data collected will be verified against the shopping receipts at the end of the trial. It will be useful to determine feasibility of this data collection approach in contest of the result of the studies using traditional grocery shopping receipt data collection methods [18].

Previous smartphone-assisted public health intervention trials have mainly focused on comparing the efficacy of technology-based intervention with traditional methods (eg, face-to-face behavior change consultations [3]). The described “smart” trials use a different approach: The FLT app is not just an intervention within a trial, but a platform enabling a fully automated RCT to be conducted. The app allows screening, consent, registration, and management of study participants remotely, without any manual input from the research team. Ethical and security requirements have also been considered during the app development, which is important, as previous systematic reviews have identified patient privacy as a potential area of concern when using smartphone technology [5].

This novel, “smart” RCT approach offers potential learning benefits for future trials, eliminating the need for in-person or telephone appointments and substantially reducing the time commitment for both researchers and trial participants. To date, limited data have been published on other trials using this approach. A study protocol by BinDhim et al [11] uses a similar “smart” RCT design. This smoking cessation trial also uses an app for screening, consenting, and randomizing trial participants, intervention delivery, and data collection. However, there are 2 key differences compared with the FLT app functionality. First, the FLT app provides a personalized intervention based on participants’ interactions with their environment, rather than delivering a generic intervention to all participants. Second, similar to the app used in the study by BinDhim et al [11], the FLT app passively records app usage and collects self-reported data via questionnaires. However, the FLT app also enables participants to actively record their food purchase data. This is of particular importance for a nutrition-labeling intervention, where there is a known shortage of outcomes based on objectively measured shopping data [27].

The FLT app design has certain limitations. Because of the relatively long duration of the projects (18 months recruitment period), it was necessary to update the app following the release of new versions of Android and Apple operating systems. Each app update required time for additional testing and resubmission to the app stores, and thus some users could experience issues with the app functionality until the update was finalized. In addition, because of the large range of smartphone devices available on the market, it was not possible to test compatibility with every one, and thus identify in advance device-specific issues. The issues affecting trial intervention delivery, such as barcode scanning or issues with producing nutrition labels, were considered of high importance for the trial, and thus were prioritized for fixing. The impact of data collection issues was partially offset by using backup data collection methods (hard copies of till receipts) [12,13]. The issues that affected the trial management process, such as very frequent reminders, created inconvenience for participants but had fewer implications for the trial outcomes.

Another limitation is that static backend food databases were used for the app. Therefore, new products appearing on the market after the app release were not recognized by the app. This may have affected both data collection and intervention delivery modes of the app, because both functions work by barcode identification within existing food databases. The impact on data collection can be managed by linking a complete trial dataset with an updated food database at a later time, and thus matching all previously unrecognized barcode numbers with new product information. However, the impact on the intervention delivery is greater, as participants cannot view nutrition labels for missing products.

Finally, the technology-based nature of the trial has a potential to contribute to sample selection bias. Although smartphone ownership is high in both Australia (up to 80% [28]) and New Zealand (up to 70% [29]), a range of factors, such as lack of an active Internet connection, may limit accessibility of some population groups to this research medium. Thus, generalizability of the smart RCT’s findings need to be further examined.

In conclusion, the FLT demonstrates the feasibility of using smartphone apps to undertake real-world nutrition-labeling interventions and enable easy collection of individualized electronic food purchasing data. The app technology allows immediate access to intervention nutrition labels in any real-world retail outlet and enables randomized comparison of the label effectiveness. The FLT app is among the first smartphone apps to enable conducting fully automated RCTs.

## Appendix

Multimedia Appendix 1 Food Label Trial app tutorial 1.

Multimedia Appendix 2 Food Label Trial app tutorial 2.

## Abbreviations

RCT: Randomized controlled trial
FLT: Food Label Trial
